# Mental health disorders among people living with human immunodeficiency virus on antiretroviral therapy in Benin: the overlooked role of sleep quality

**DOI:** 10.7189/jogh.16.04076

**Published:** 2026-03-06

**Authors:** Boni Maxime Ale, Calixte Oswald Assogba, Eugénie Dansou, Olushina Ayo Junior Ale, Oswald Lionel Koutangni, Adébiyi Raphaël K Alogou, Simon Giscard Akpi, Kenneth Geovania Dèlonou Damassoh, Eric Youm, Nelly Njeri Wakaba, Houénoudé Mickaël Arnaud Assogba, Kouessi Anthelme Agbodande, Angèle Azon Kouanou, Franck Biaou Guy Ale

**Affiliations:** 1 Global Mental Health Research Center, Holo Global Health Research Institute, Cotonou, Benin; 2 Health Data Acumen, Cotonou, Benin; 3 Institute of Tropical and Infectious Diseases, University of Nairobi, Nairobi, Kenya; 4 School of Public Health, Moi University, Eldoret, Kenya; 5 Laboratoire des Maladies Chroniques et Neurologiques, Abomey Calavi, Benin; 6 Centre National Hospitalier et Universitaire Hubert Koutoucou Maga, Cotonou, Benin; 7 Faculty of Health Sciences, University of Abomey Calavi, Abomey Calavi, Benin

## Abstract

**Background:**

Depression and anxiety are common comorbidities among people living with HIV (PLHIV) and may be influenced by sleep quality; evidence from Benin remains limited. We assessed the prevalence of depression and anxiety among PLHIV on antiretroviral therapy (ART) and evaluated their association with sleep quality.

**Methods:**

We conducted a hospital-based cross-sectional study at Benin’s National Teaching Hospital from December 2023 to February 2024. Adults on ART ≥ 6 months were randomly sampled from the clinic registry. Validated tools were used: Patient Health Questionnaire-9 and Generalized Anxiety Disorder-7, moderate-or-worse threshold ≥ 10; Pittsburgh Sleep Quality Index, poor sleep > 5. Multivariable logistic regression identified factors independently associated with depression and anxiety.

**Results:**

Among 312 participants (68.3% female; mean age 44.3 ± 12.3 years), the prevalence of depression and anxiety was 20.8% (n/N = 65/312) and 12.8% (n/N = 40/312), respectively; poor sleep quality was common (57.1%, n/N = 178/312). Good sleep quality was independently protective for both depression with adjusted odds ratio (aOR) = 0.3, 95% confidence interval (CI) = 0.2–0.6 and anxiety (aOR = 0.4; 95% CI = 0.2–0.9). Age ≥ 44 years was associated with higher odds of anxiety (aOR = 2.1; 95% CI = 1.0–4.5), while other sociodemographic and clinical covariates were not independently associated.

**Conclusions:**

Moderate-or-worse depressive and anxiety symptoms are frequent among PLHIV in this setting, and sleep quality shows a robust independent association with both outcomes. Integrating routine mental-health and sleep screening with clear referral pathways within ART services is warranted; longitudinal and interventional studies should test whether improving sleep reduces symptoms and enhances ART outcomes.

HIV/AIDS affects 38.4 million people living with HIV (PLHIV) worldwide as of 2021. Notably, two-thirds of this population reside in sub-Saharan Africa, where the disease burden is disproportionately high [1]. Over the past few decades, significant advancements in the management of HIV/AIDS, particularly through the development of potent antiretroviral therapy (ART), have transformed HIV infection from an acute, life-threatening disease into a chronic condition [2–5]. While this transformation has improved life expectancy and reduced morbidity, it has also redefined the clinical and public health challenges associated with HIV, particularly in the domain of mental health comorbidities. Among these, anxiety and depression are notably prevalent, exerting profound effects on treatment adherence, disease progression, and overall quality of life among PLHIV [6].

A growing body of evidence highlights the high prevalence of anxiety and depression among individuals living with HIV, with estimates varying across different settings. For instance, studies indicate a prevalence ranging from 13.8% in Guinea to 79.0% in China, underscoring both geographic and systemic variations in mental health burden and care accessibility [2,6–8]. These psychiatric conditions are often underdiagnosed and undertreated, particularly in resource-limited settings such as sub-Saharan Africa, where mental health services are scarce [9,10]. The lack of adequate screening and treatment for psychiatric comorbidities among PLHIV not only exacerbates individual suffering but also contributes to poorer health outcomes, increasing the risk of treatment non-adherence, disease progression, and even mortality [9,11].

Several sociodemographic and clinical factors have been identified as significant determinants of anxiety and depression among PLHIV. Socioeconomic disparities, particularly low levels of education and financial instability, have been strongly linked to increased psychological distress in this population [11]. Additionally, the female sex has been associated with a higher likelihood of developing anxiety and depression. Furthermore, poor sleep quality has been recognised as a crucial factor contributing to the onset and exacerbation of psychiatric symptoms, with studies demonstrating a strong correlation between sleep disturbances and the severity of depression and anxiety in PLHIV [11]. Biologically, sleep disturbances trigger dysregulation of the hypothalamic-pituitary-adrenal axis and increase pro-inflammatory cytokine levels (*e.g.* interleukin-6, tumour necrosis factor alpha), which are strongly linked to the onset and persistence of depression and anxiety [12,13]. Sleep impairment also disrupts circadian regulation of mood and cognitive processes, thereby exacerbating vulnerability to psychiatric symptoms [14]. In the context of HIV, poor sleep can further undermine adherence to ART, as fatigue and low motivation reduce consistent medication intake, which in turn worsens virological outcomes and heightens psychological distress [15]. These interrelated biological and behavioural pathways underscore the plausibility of poor sleep acting both as a contributor to and a consequence of mental health disorders among PLHIV.

The implications of untreated mental health disorders in PLHIV extend beyond the individual level, affecting treatment adherence and overall public health outcomes. Depression and anxiety have been shown to significantly reduce adherence to ART, which is crucial for achieving and maintaining viral suppression [2,16–18]. Individuals experiencing mental health distress may struggle with motivation, forgetfulness, and feelings of hopelessness, leading to suboptimal medication adherence and higher risks of virological failure [17,19]. Additionally, the presence of psychiatric disorders is associated with an increased risk of suicidality, further compounding the health risks for affected individuals [2]. Despite the high burden of anxiety and depression among PLHIV, access to mental health screening and treatment remains alarmingly low. This underscores an urgent need for policies and interventions aimed at enhancing mental health integration within HIV care programmes [20]. In Benin, for instance, there is no report assessing the association of mental health and sleep disorders. Considering these challenges, we hypothesised that poor sleep quality is independently associated with depression and anxiety in PLHIV after adjusting for sociodemographic and clinical factors. Thus, our study seeks to assess the prevalence of anxiety and depression and associated factors, including the impact of poor sleep, among PLHIV receiving ART, to inform the integration of sleep and mental health screening into HIV care in Benin.

## METHODS

### Study setting and design

This was a hospital-based cross-sectional study assessing the prevalence of anxiety and depression and factors associated, carried out at Benin’s National Teaching Hospital Hubert Koutoukou Maga (CNHU/HKM) conducted between December 2023–February 2024.

### Study population

The study population was composed of people living with HIV on antiretroviral treatment followed up in the outpatient clinic of Benin’s National Teaching Hospital.

### Inclusion and exclusion criteria

We included patients aged over 18 and on ART for more than six months. Patients with severe cognitive disorders preventing them from responding to questionnaires, those undergoing psychiatric treatment for other chronic psychiatric conditions, pregnant women, or women in the postpartum period were excluded.

### Sample size and sampling techniques

The sample size was estimated using Schwarz’s formula, based on 13.8% prevalence of anxiety, 16.9% prevalence of depression [11], a 5% standard error margin and with a 95% confidence interval (CI). To account for potential non-respondents, an additional 10% was added, which led to the total minimal sample size of 203 for anxiety and 238 for depression. Participants were selected through a simple random sampling method using a random generator in Excel applied to the hospital’s database of actively followed-up patients.

### Study variables

The primary outcomes were anxiety and depression, and the key independent variable was sleep quality (poor or good quality). The other independent variables were sociodemographic (age, sex, level of education, marital status, professional status), medical (comorbidities, hypertension, cardiovascular diseases), and HIV-associated factors (ART treatment duration, viral load, treatment protocol).

### Operational definition

Anxiety was defined using the Generalized Anxiety Disorder-7 (GAD-7) scale. We categorised anxiety symptoms as none/minimal (0–4), mild (5–9), moderate (10–14), and severe (15–21), with primary analyses using a ‘moderate-or-worse’ threshold of ≥ 10. This classification follows established guidelines for assessing anxiety disorders in clinical and research settings [10].

Depression was defined according to the Patient Health Questionnaire-9 (PHQ-9) scale. We categorised depressive symptoms as none/minimal (0–4), mild (5–9), moderate (10–14), moderately severe (15–19), and severe (20–27), with primary analyses using a ‘moderate-or-worse’ threshold of ≥10 [21]. This threshold is widely used for detecting depressive symptoms in PLHIV.

Quality of sleep was evaluated using the Pittsburgh Sleep Quality Index (PSQI), with a global score > 5 indicating poor sleep quality [22].

Antiretroviral therapy duration was defined as the time since the first prescription of ART, based on the patient's medical records.

Alcohol consumption was defined based on patient declarations, with current alcohol use referring to any alcohol consumption in the past month.

Smoking status was classified as current smoker (any cigarette use in the past month), former smoker (stopped smoking for more than a month), or never smoker based on self-reported history.

Education level was classified as no formal education, primary, secondary, or higher.

Employment status was classified as employed or unemployed.

Hypertension was defined according to the European Society of Hypertension guidelines as a systolic blood pressure ≥140 mm Hg and/or diastolic blood pressure ≥ 90 mm Hg [23], or/and the current use of antihypertensive medication as documented in medical records.

Body mass index (BMI) was used to classify physical status as follows: underweight: BMI ≤ 18.5 kg/m^2^, normal weight: BMI between 18.5–24.9 kg/m^2^, overweight: BMI between 25.0–29.9 kg/m^2^, obesity: BMI > 30 kg/m^2^.

Comorbidities such as diabetes and cardiovascular diseases were documented based on patients' medical records and self-reported diagnoses confirmed by health care providers.

### Data collection

#### Data quality control and collection procedure

Eligible participants were contacted via phone by hospital staff, who provided a detailed explanation of the study’s objectives and procedures. Patients who agreed to participate were scheduled for an appointment at the clinic, where they provided informed consent before data collection commenced. incorporating validated evaluation tools such as the PSQI, STOP-BANG Questionnaire, PHQ-9, and GAD-7. The data were sourced from multiple channels, including patient self-reports, medical records, and physical measurements. Trained staff conducted face-to-face interviews with written informed consent.

#### Data processing and analyses

To ensure high data quality, the research team underwent comprehensive training before data collection. The data collection process was digitised using the Open Data Kit software on smartphones, enabling real-time monitoring for completeness and accuracy. Following the collection, data were consolidated, reviewed, and validated for internal consistency. The collected data were analysed using *R* software version 4.4.3 (Vienna, Austria). The analysis comprised descriptive statistics, with results presented as means, standard deviations, and percentages; bivariate analysis, employing the χ^2^ test for categorical variables and the Wilcoxon test for continuous variables, to examine associations between independent and dependent variables, and multivariate analysis, conducted through logistic regression, to identify factors independently associated with anxiety and depression among PLHIV on ART.

### Ethics approval and consent to participate

The study sought the approval of the Health Research Ethics Committee of the Cotonou Faculty of Health Sciences and the Local Biomedical Research Ethics Committee of the University of Parakou. In addition, the study sought the authorisation of the administration of the CNHU-HKM of Cotonou on the one hand and of the National Reference Center for Research and Care for HIV (CNRRPEC) on the other hand to allow data collection in the institution.

Written informed consent was obtained from all the subjects before the study. The study was also conducted following the Helsinki Declaration of research involving human subjects.

## RESULTS

A total of 312 PLHIV on ART were included; 213 (68.3%) were female, and 160 (51.3%) were aged ≥44 years. Educational attainment was low for many participants (no formal education: n = 84, 26.9%). Most were employed (n = 253, 81.1%), 25 (8.0%) reported current tobacco use, 132 (42.3%) reported alcohol use, and 271 (86.9%) engaged in regular physical activity. Hypertension affected 91 participants (29.2%) and cardiovascular disease 18 (5.8%). By BMI, 28 (9.0%) were underweight, 140 (44.9%) normal weight, 77 (24.7%) overweight, and 67 (21.5%) obese. Antiretroviral therapy duration exceeded five years for 190 participants (60.9%); 271 (86.9%) were on Dolutegravir/lamivudine/tenofovir (TDF-3TC-DTG) and 31 (9.9%) on Efavirenz/lamivudine/tenofovir (EFV/3TC/TDF). Nearly all had HIV-1 (n = 308, 98.7%).

World Health Organization clinical stage distribution was (out of 312 participants):

• Stage 1: 57 (18.3%)

• Stage 2: 61 (19.6%)

• Stage 3: 152 (48.7%)

• Stage 4: 42 (13.5%).

Virologic failure was uncommon (n = 8, 2.6%), as were clinical (n = 5, 1.6%) and immunologic (n = 1, 0.3%) failures. Poor sleep quality (PSQI>5) affected 178 participants (57.1%) (**Table 1**).

**Table 1:** Characteristics of people living with HIV on ART in Benin’s National Teaching Hospital, 2024

Characteristics	n	%
Mean age	44.3	12.3
Age group		
*Under 44 y*	152	48.7
*Over 44 y*	160	51.3
Sex		
*Male*	99	31.7
*Female*	213	68.3
Level of education		
*No formal education*	84	26.9
*Primary school*	80	25.6
*Secondary school*	93	29.8
*University*	55	17.6
Marital status		
*Not married*	124	39.7
*Married*	188	60.3
Employment status		
*Unemployed*	59	18.9
*Employed*	253	81.1
Tobacco consumption		
*Non-smoker*	287	92.0
*Smoker*	25	8.0
Alcohol consumption		
*Non-drinker*	180	57.7
*Drinker*	132	42.3
Time since diagnosis		
*Less than 5 y*	95	30.4
*5 y or more*	217	69.6
Time since initiation of ART		
*Less than 5 y*	95	30.4
*5 y or more*	217	69.6
ART regimen		
*ABC-3TC-DTG*	4	1.3
*Others*	1	0.3
*AZT+3TC+EFV*	2	0.6
*AZT+3TC+NVP*	1	0.3
*TDF-3TC-DTG*	271	86.9
*TDF+3TC+EFV*	31	9.9
*TDF+3TC+NVP*	2	0.6
HIV type		
*HIV 1*	308	98.7
*HIV 1 and 2*	3	1.0
*HIV 2*	1	0.3
Viral load (copies)		
*Less than 5*	242	77.6
*5–500*	46	14.7
*501–1000*	3	1.0
*More than 1000*	21	6.7
WHO Stage		
*Stage 1*	57	18.3
*Stage 2*	61	19.6
*Stage 3*	152	48.7
*Stage 4*	42	13.5
Hypertension		
*No*	247	79.2
*Yes*	65	20.8
Body mass index		
*Underweight*	28	9.0
*Normal weight*	140	44.9
*Overweight*	77	24.7
*Obese*	67	21.5
Regular physical activity		
*No*	41	13.1
*Yes*	271	86.9
Sleep quality		
*Poor quality*	178	57.1
*Good quality*	134	42.9

ART – antiretroviral therapy, WHO – World Health Organization, ABC-3TC-DTG – Abacavir/Lamivudine/Dolutegravir, AZT + 3TC + EFV – Zidovudine + Lamivudine + Efavirenz, AZT + 3TC + NVP – Zidovudine + Lamivudine + Nevirapine, TDF-3TC-DTG – Tenofovir disoproxil fumarate + Lamivudine + Dolutegravir, TDF + 3TC + EFV – Tenofovir disoproxil fumarate + Lamivudine + Efavirenz, TDF + 3TC + NVP – Tenofovir disoproxil fumarate + Lamivudine + Nevirapine

**Figure 1:**
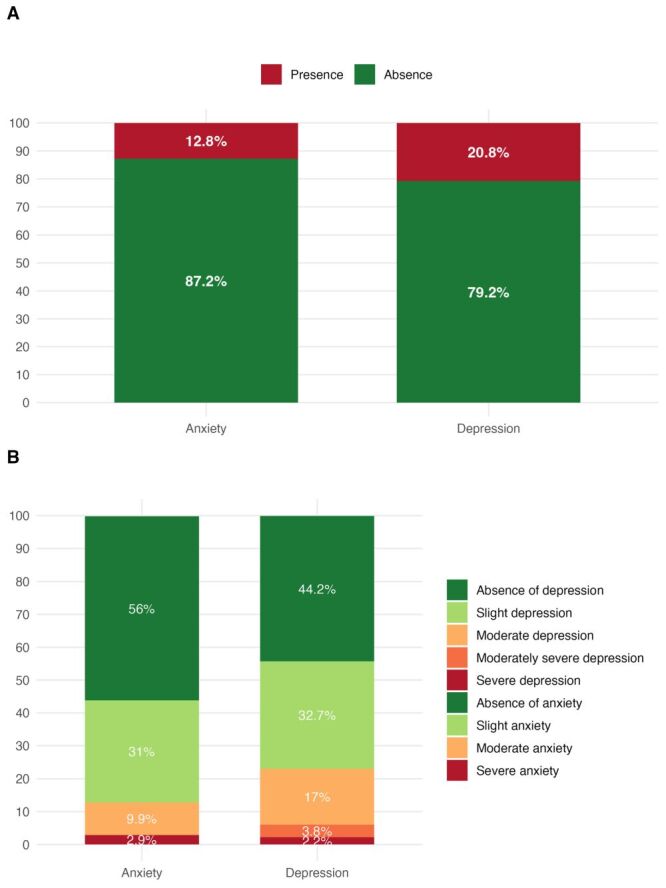
Prevalence of anxiety and depression in people living with HIV in Benin’s National Teaching Hospital, 2024. **Panel A.** Overall prevalence of anxiety and depression. **Panel B.** Distribution of anxiety and depression severity levels among participants.

Mental health burden was substantial: 65 participants (20.8%) met criteria for depression. Symptom severity was distributed as follows: slight (n = 102, 32.7%), moderate (n = 53, 17.0%), moderately severe (n = 12, 3.8%), and severe (n = 7, 2.2%). Anxiety criteria were met by 40 participants (n = 12.8%); severity levels included slight symptoms in 98 (31.0%), moderate in 31 (9.9%), and severe in 9 (2.9%) (**Figure 1**, Panels A–B). Poor sleep quality (PSQI>5) was common overall (n/N = 178/312, 57.1%) and more frequent among those with depression (n/N = 50/65, 76.9%) *vs*. those without (n/N = 128/247, 51.8%), and among those with anxiety (n/N = 30/40, 75.0%) *vs*. those without (n/N = 148/272, 54.4%). In multivariable models, good sleep quality remained independently protective for both depression (aOR = 0.3; 95% CI = 0.2–0.6, *P* < 0.001) and anxiety (aOR = 0.4; 95% CI = 0.2–0.9, *P* = 0.032), implying roughly 3-fold higher odds of depression (**Table 2**) and 2.5-fold higher odds of anxiety with poor sleep (**Table 3**). For anxiety, age ≥44 years was also associated with higher odds (aOR = 2.1; 95% CI = 1.0–4.5, *P* = 0.045). The association between being married and lower odds of depression (aOR = 0.6; 95% CI = 0.3–0.8) was observed, though the *P*-value approached but did not reach conventional significance (*P* = 0.087). Other sociodemographic and clinical variables were not independently associated after adjustment. We did not analyse sleep or mental-health outcomes by ART regimen because too few participants received regimens other than TDF-3TC-DTG to permit meaningful inference.

**Table 2:** Associated factors of depression in people living with HIV on ART in Benin’s National Teaching Hospital, 2024

Characteristics		Unadjusted model				Adjusted model	
	**n**	**Absence (n, %)**	**Presence (n, %)**	**OR (95% CI)**	***P*-value**	**aOR (95% CI)**	***P*-value**
		**N = 247**	**N = 65**				
Sleep quality	312				<0.001		<0.001
*Poor quality*		128 (51.8)	50 (76.9)	Ref		Ref	
*Good quality*		119 (48.2)	15 (23.1)	0.3 (0.2–0.6)		0.3 (0.2–0.6)	
Age group	312				0.710		
*Less than 44*		119 (48.2)	33 (50.8)	Ref			
*44 and over*		128 (51.8)	32 (49.2)	0.9 (0.5–1.6)			
Sex	312				0.278		
*Male*		82 (33.2)	17 (26.2)	Ref			
*Female*		165 (66.8)	48 (73.8)	1.4 (0.8–2.7)			
Level of education	312				0.947		
*No formal education*		68 (27.5)	16 (24.6)	Ref			
*Primary school*		63 (25.5)	17 (26.2)	1.1 (0.5–2.5)	0.725		
*Secondary school*		72 (29.1)	21 (32.3)	1.2 (0.6–2.6)	0.564		
*University*		44 (17.8)	11 (16.9)	1.1 (0.4–2.5)	0.890		
Marital status	312				0.141		0.087
*Not married*		93 (37.7)	31 (47.7)	Ref		Ref	
*Married*		154 (62.3)	34 (52.3)	0.7 (0.4–1.2)		0.6 (0.3–0.8)	
Employment status	312				0.543		
*Unemployed*		45 (18.2)	14 (21.5)	Ref			
*Employed*		202 (81.8)	51 (78.5)	0.8 (0.4–1.6)			
Tobacco consumption	312				0.915		
*Non-smoker*		227 (91.9)	60 (92.3)	Ref			
*Smoker*		20 (8.1)	5 (7.7)	0.9 (0.3–2.4)			
Alcohol consumption	312				0.672		
*Non-drinker*		141 (57.1)	39 (60.0)	Ref			
*Drinker*		106 (42.9)	26 (40.0)	0.9 (0.5–1.5)			
Use of recreational substances	312				0.111		0.169
*No*		246 (99.6)	63 (96.9)	Ref		Ref	
*Yes*		1 (0.4)	2 (3.1)	7.8 (0.7–169.6)		5.5 (0.5–121.7)	
Regular physical activity	312				0.547		
*No*		31 (12.6)	10 (15.4)	Ref			
*Yes*		216 (87.4)	55 (84.6)	0.8 (0.4–1.8)			
Hypertension	312				0.597		
*No*		194 (78.5)	53 (81.5)	Ref			
*Yes*		53 (21.5)	12 (18.5)	0.8 (0.4–1.6)			
History of cardiovascular events	312				0.070		
*No*		236 (95.5)	58 (89.2)	Ref			
*Yes*		11 (4.5)	7 (10.8)	2.6 (0.9–6.9)			
Body mass index	312				0.664		
*Underweight*		23 (9.3)	5 (7.7)	Ref			
*Normal weight*		107 (43.3)	33 (50.8)	1.4 (0.5–4.5)	0.511		
*Overweight*		61 (24.7)	16 (24.6)	1.2 (0.4–4.0)	0.741		
*Obese*		56 (22.7)	11 (16.9)	0.9 (0.3–3.1)	0.864		
Duration on ART	312				0.714		
*Less than 5 y*		74 (30.0)	21 (32.3)	Ref			
*5 y or more*		173 (70.0)	44 (67.7)	0.9 (0.5–1.6)			
Type of HIV	312				0.193		
*HIV 1*		245 (99.2)	63 (96.9)	Ref			
*HIV 1 and 2*		1 (0.4)	2 (3.1)	7.8 (0.7–168.9)	0.096		
*HIV 2*		1 (0.4)	0 (0.0)	0.0	0.988		
WHO stage of HIV	312				0.038		
*Stage 1*		47 (19.0)	10 (15.4)	Ref			
*Stage 2*		55 (22.3)	6 (9.2)	0.5 (0.2–1.5)	0.227		
*Stage 3*		111 (44.9)	41 (63.1)	1.7 (0.8–3.9)	0.161		
*Stage 4*		34 (13.8)	8 (12.3)	1.1 (0.4–3.1)	0.848		

aOR – adjusted odds ratio, ART – antiretroviral therapy, CI – confidence interval, WHO – World Health Organization

**Table 3:** Associated factors of anxiety in people living with HIV on ART in Benin’s National Teaching Hospital, 2024

Characteristics	Unadjusted model					Adjusted model	
	**n**	**Absence, n (%)**	**Presence, n (%)**	**OR (95% CI)**	***P*-value**	**aOR (95% CI)**	***P*-value**
		**N = 272**	**N = 40**				
Sleep Quality	312				0.014		0.032
*Poor quality*		148 (54.4)	30 (75.0)	Ref		Ref	
*Good quality*		124 (45.6)	10 (25.0)	0.4 (0.2–0.8)		0.4 (0.2–0.9)	
Age group	312				0.028		0.045
*Less than 44*		139 (51.1)	13 (32.5)	Ref		Ref	
*44 and over*		133 (48.9)	27 (67.5)	2.2 (1.1–4.5)		2.1 (1.0–4.5)	
Sex	312				0.538		0.666
*Male*		88 (32.4)	11 (27.5)	Ref		Ref	
*Female*		184 (67.6)	29 (72.5)	1.3 (0.6–2.7)		1.2 (0.6–2.7)	
Level of education	312				0.066		
*No formal education*		68 (25.0)	16 (40.0)	Ref			
*Primary school*		73 (26.8)	7 (17.5)	0.4 (0.1–1.0)	0.063		
*Secondary school*		79 (29.0)	14 (35.0)	0.8 (0.3–1.7)	0.480		
*University*		52 (19.1)	3 (7.5)	0.2 (0.1–0.8)	0.032		
Marital status	312				0.511		
*Not married*		110 (40.4)	14 (35.0)	Ref			
*Married*		162 (59.6)	26 (65.0)	1.3 (0.6–2.6)			
Employment status	312				0.499		
*Unemployed*		53 (19.5)	6 (15.0)	Ref			
*Employed*		219 (80.5)	34 (85.0)	1.4 (0.6–3.8)			
Tobacco consumption	312				0.754		
*Non-smoker*		249 (91.5)	38 (95.0)	Ref			
*Smoker*		23 (8.5)	2 (5.0)	0.6 (0.1–2.0)			
Alcohol consumption	312				0.979		
*Non-drinker*		157 (57.7)	23 (57.5)	Ref			
*Drinker*		115 (42.3)	17 (42.5)	1.0 (0.5–2.0)			
Use of recreational substances	312				0.044		0.113
*No*		271 (99.6)	38 (95.0)	Ref		Ref	
*Yes*		1 (0.4)	2 (5.0)	14.3 (1.3–311.3)		7.3 (0.7–162.9)	
Regular physical activity	312				0.258		
*No*		38 (14.0)	3 (7.5)	Ref			
*Yes*		234 (86.0)	37 (92.5)	2.0 (0.7–8.6)			
Hypertension	312				0.889		
*No*		215 (79.0)	32 (80.0)	Ref			
*Yes*		57 (21.0)	8 (20.0)	0.9 (0.4–2.1)			
History of cardiovascular events	312				0.487		
*No*		255 (93.8)	39 (97.5)	Ref			
*Yes*		17 (6.3)	1 (2.5)	0.4 (0.0–2.0)			
Body mass index	312				0.002		
*Underweight*		26 (9.6)	2 (5.0)	Ref			
*Normal weight*		115 (42.3)	25 (62.5)	2.8 (0.8–18.2)	0.175		
*Overweight*		65 (23.9)	12 (30.0)	2.4 (0.6–16.1)	0.273		
*Obese*		66 (24.3)	1 (2.5)	0.2 (0.0–2.1)	0.192		
Duration on ART	312				0.242		
*Less than 5 y*		86 (31.6)	9 (22.5)	Ref			
*5 y or more*		186 (68.4)	31 (77.5)	1.6 (0.8–3.7)			
Type of HIV	312				>0.999		
*HIV 1*		268 (98.5)	40 (100.0)	Ref			
*HIV 1 and 2*		3 (1.1)	0 (0.0)	0.0	0.992		
*HIV 2*		1 (0.4)	0 (0.0)	0.0	0.995		
WHO stage of HIV	312				0.095		
*Stage 1*		53 (19.5)	4 (10.0)	Ref			
*Stage 2*		50 (18.4)	11 (27.5)	2.9 (0.9–11.1)	0.083		
*Stage 3*		129 (47.4)	23 (57.5)	2.4 (0.9–8.3)	0.129		
*Stage 4*		40 (14.7)	2 (5.0)	0.7 (0.1–3.6)	0.644		

aOR – adjusted odds ratio, ART – antiretroviral therapy, CI – confidence interval, WHO – World Health Organization

## DISCUSSION

This study aimed to assess the prevalence of depression and anxiety and their association with sleep quality among PLHIV on ART in Benin. Out of 312 PLHIV at CNHU-HKM, 65 (20.8%) had depression (mild: n = 53, 17.0%; severe: n = 7, 2.2%) and 40 (12.8%) had anxiety (mild: n = 31, 9.9%; severe: n = 9, 2.9%), underscoring a meaningful mental-health burden. In multivariable analyses, good sleep quality was protective for both outcomes (depression aOR = 0.3; 95% CI = 0.2–0.6; anxiety aOR = 0.4; 95% CI = 0.2–0.9), implying that poor sleep was associated with roughly 3-fold (depression) and 2.5-fold (anxiety) higher odds. Age ≥44 years increased the odds of anxiety (aOR = 2.1; 95% CI = 1.0–4.5), marriage showed a protective trend for depression (aOR = 0.6; *P* = 0.087), and sex was not independently associated after adjustment.

The prevalence of depression observed in our study (20.8%) surpassed previously reported rates in Nigeria (14.3%) [24]. This highlights the urgent need for targeted interventions to address mental health issues in PLHIV, particularly in sub-Saharan Africa, where access to psychological care remains limited. While certain studies have reported even higher depression rates in France (28.1%) and China (79%), the small sample size in these investigations limits generalisability [2,8]. These findings reinforce the notion that depression prevalence varies significantly across different populations, depending on cultural, socioeconomic, and health care accessibility factors. Nonetheless, the consistently high prevalence of depression among PLHIV across studies is a major cause for concern [20].

Similarly, the prevalence of anxiety (12.8%) in our study is notably high as previously reported in West Africa. For instance, Camara et al. (2020) reported anxiety prevalence (13.8%) in Guinea, while Tesfaw et al. documented an even higher rate (32.4%) [7,25]. Despite these variations, our study confirms that PLHIV are disproportionately affected by mental health disorders, with higher anxiety rates than those observed in the general population [26]. These rates may be higher because the mental-health system is thin; the country reports 0.43 mental-health workers per 100 000 and sub-optimal integration of mental health into primary care; limiting detection and treatment in ART clinics [27]. Socioeconomic stressors are substantial and food insecurity is linked to worse mental health among PLHIV [28]. HIV-related stigma documented in the region deters help-seeking, while routine mental-health screening is not yet standard in many HIV programmes.

Our study population had a strong female predominance, which aligns with findings from similar studies conducted in Benin, Nigeria, Rwanda, and Guinea [6,7,24]. However, this trend contrasts with observations in France, where male predominance was reported [2]. This sex disparity in African countries may be attributed to increased HIV screening among women during maternity care programmes, leading to a higher diagnosis rate in females compared to males. Despite the absence of an independent association between sex and mental health disorders in our study, it is well documented that women are biologically and socially more vulnerable to developing anxiety and depression. For instance, Turk et al. highlighted hormonal differences as key contributors to this disparity [29].

Hypertension was observed in 29.2% of our study population, a prevalence comparable to findings from prior studies in Benin and Guinea [7,30,31]. However, this rate is slightly lower than that reported by Feuillet et al. in France, likely reflecting differences in lifestyle, diet, and health care access [2]. Furthermore, obesity prevalence in our cohort was lower than reported in PLHIV in France, which may be attributable to nutritional and environmental variations. These findings suggest that noncommunicable diseases such as hypertension and obesity are increasingly becoming a concern among PLHIV, emphasising the importance of comprehensive HIV care that includes metabolic and cardiovascular health monitoring.

In adjusted analyses, age over 44 years was associated with higher odds of anxiety (aOR = 2.1; 95% CI = 1.0–4.5). This age effect is biologically and contextually plausible: chronic immune activation and inflammation persist despite ART and have been linked to neuroinflammation and affective symptoms in PLHIV, providing a substrate for anxiety in later life [32,33]. Ageing is also accompanied by lighter, more fragmented sleep and a greater burden of sleep disorders, which show robust associations with anxiety; moreover, insomnia itself predicts subsequent anxiety disorders [34]. Among women, the menopausal transition adds sleep disruption and mood symptoms, with similar patterns reported in cohorts of women living with HIV [35].

Beyond the high burden observed, our data show a robust, independent association between sleep quality and mental health: good sleep quality was protective for both depression (aOR = 0.3; 95% CI = 0.2–0.6) and anxiety (aOR = 0.4; 95% CI = 0.2–0.9), implying roughly 3-fold higher odds of depression and about 2.5-fold higher odds of anxiety among participants with poor sleep; this pattern was reflected in prevalence contrasts and persisted after adjustment for sociodemographic and clinical covariates. These findings are biologically and behaviourally plausible: sleep disturbance activates inflammatory pathways and hypothalamic-pituitary-adrenal (HPA) axis dysregulation linked to depressive and anxious symptomatology [12–14], and poorer sleep is associated with antiretroviral non-adherence that can worsen virologic control and psychological distress [15]. HIV-specific evidence also connects sleep impairment with heightened anxiety and depressive symptoms, particularly among women living with HIV [11]. Nonetheless, the cross-sectional design precludes causal inference, and unmeasured factors such as pain/neuropathy, stigma, and food insecurity may contribute. Antiretroviral therapy regimen effects could not be assessed given the predominance of TDF-3TC-DTG. Taken together, our results support routine PSQI-based screening and brief sleep interventions such as sleep-hygiene counselling, cognitive behavioural therapy for insomnia referral pathways within ART clinics as pragmatic strategies to mitigate mental-health morbidity in this population [36].

## CONCLUSIONS

We identified a substantial burden of depression and anxiety among PLHIV on ART and a robust, independent association with sleep quality, with good sleep protective for both outcomes. After adjustment, age ≥44 years increased the odds of anxiety, marriage showed a protective trend for depression, and sex and other clinical covariates were not independently associated. These findings support integrating routine PHQ-9/GAD-7 and PSQI screening into ART services with clear referral pathways (*e.g.* Cognitive Behavioral Therapy for Insomnia) and brief psychosocial care. Key limitations include the cross-sectional, single-centre design and regimen homogeneity; prospective and interventional studies should assess whether improving sleep reduces symptoms and enhances ART adherence and virologic outcomes.
